# Deep-sea Microbial Dataset of the Antarctic Ocean (dsMDAO): A high-resolution deep-sea microbial dataset of the Antarctic Ocean reveals potential for taxonomic and functional diversity

**DOI:** 10.1099/mgen.0.001847

**Published:** 2026-10-05

**Authors:** Xingang Song, Shulei Jia, Lingling Chen, Chengjuan Cao, Zhongzhen Chen, Lingxiao Hang, Huihuang Jiang, Zhongwei Chen

**Affiliations:** 1 Nantong Marine Center, Ministry of Natural Resources, Nantong, 226002, PR China; 2 School of Basic Medical Sciences, Tianjin Medical University, Tianjin, 300070, PR China; 3 Hebei Carbon Investment Technology Development Co. Ltd, Shijiazhuang, 050000, PR China

**Keywords:** Antarctic deep-sea microbiome, functional gene dataset, metagenome-assembled genomes (MAGs), microbial diversity

## Abstract

Antarctic deep-sea ecosystems harboured unique and metabolically versatile micro-organisms that sustained key biogeochemical processes under extreme polar conditions. However, the genomic diversity and ecological functions of these sedimentary or deep-sea microbial communities remained largely unexplored. Here, we constructed a comprehensive deep-sea microbial dataset of the Antarctic Ocean (dsMDAO) by integrating three newly sequenced Prydz Bay sediment metagenomes with 22 publicly available datasets (9 seawater and 13 sediment samples), spanning water depths of ~300–3500 m. Genome binning yielded 186 metagenome-assembled genomes spanning 19 phyla, including 175 bacteria and 11 archaea, a substantial proportion of which represent previously uncharacterized species. Meanwhile, reads mapping with the available standard Kraken2 database (k2_standard) enabled the expansion of species richness of the dsMDAO database. Furthermore, functional annotation revealed diverse metabolic and ecological potentials, including carbon, nitrogen and sulphur cycling, as well as secondary metabolite biosynthesis, virulence-associated defence and cold-adaptation mechanisms. Sediment microbiomes exhibited higher phylogenetic and functional diversity, enriched in *Thaumarchaeota* and *Chloroflexi*, whereas seawater communities were dominated by *Proteobacteria* with more competitive biosynthetic and interaction potentials. Co-occurrence analyses further indicated complex and competitive networks in seawater versus modular and cooperative assemblages in sediments, reflecting distinct ecological strategies. Collectively, dsMDAO provides the first genome-resolved dataset of Antarctic deep-sea microbiota, revealing the hidden taxonomic and functional diversity that underpins ecosystem resilience in polar oceans. This resource lays a foundation for future ecological, evolutionary and biotechnological exploration of Antarctic microbial dark matter.

**Impact Statement**

This study presents a deep-sea microbial dataset of the Antarctic Ocean, the first genome-resolved microbial dataset of the Antarctic deep sea. By integrating new and public metagenomic data, it uncovers hidden taxonomic and functional diversity, including cold adaptation, nutrient cycling and bioactive compound potential, offering a critical resource for polar ecology, climate science and biotechnology.

## Data Summary

The raw sequencing reads generated in this study have been deposited in the NCBI Sequence Read Archive (SRA) under BioProject accession number PRJNA1267735 (https://www.ncbi.nlm.nih.gov/bioproject/PRJNA1267735/).

The predicted antimicrobial peptides, identified using AMPShpere from the Antarctic deep-sea metagenomic datasets, are available in the dedicated GitHub repository (https://github.com/xielisos567/dsMDAO_AMPs) and have been archived with Zenodo under DOI: 10.5281/zenodo.22235425. This repository contains the complete annotation results (Antarctic_AMPs.xlsx) and the AMPShpere output files (AMPShpere.zip).

The assembled metagenome-assembled genomes and the corresponding functional annotation results can be accessed at https://github.com/xielisos567/dsMDAO_MAGs/releases/tag/v1.0.0.

All external data and code are publicly accessible. Custom analysis scripts are available from the corresponding author upon reasonable request.

## Introduction

Antarctic marine ecosystems, particularly those surrounding Antarctica, represent one of the most extreme and least explored microbial frontiers on Earth [1]. The Antarctic Ocean, covering nearly 10% of the global ocean surface, functions as a key regulator of global biogeochemical processes, including carbon sequestration, nutrient cycling and climate feedback mechanisms. These high-latitude ecosystems are characterized by persistently low temperatures, extreme seasonality, oligotrophic water and extensive sea-ice coverage that together generate strong physical and chemical gradients [2–4]. Microbial communities thriving under these conditions are not only the primary drivers of local biogeochemical transformations but also serve as critical sentinels of environmental change in a rapidly warming Antarctic region [5]. The study of these micro-organisms, therefore, is central to understanding how life adapts to extreme conditions and how microbial processes modulate global elemental cycles in response to environmental perturbations [1, 6, 7].

Previous studies across Antarctic marine habitats, including sea ice [8], coastal waters [9, 10], pelagic waters [11] and sediments [12], have revealed pronounced spatial and seasonal variation in microbial community composition and have highlighted the central roles of micro-organisms in carbon fixation and remineralization, nitrogen transformation and sulphur cycling [5, 13]. However, despite these extensive ecological investigations, our understanding of Antarctic micro-organisms remains largely limited at the community level, while genome-resolved insights into their diversity, functions and evolutionary adaptations are still unevenly distributed across environments and geographic regions [13, 14]. In particular, deep-sea microbial communities have received comparatively limited genome-resolved characterization, constraining our understanding of their taxonomic novelty, metabolic capacities, ecological interactions and potential contributions to biogeochemical cycling [13]. Early research in Antarctic microbiology relies heavily on culture-dependent techniques, which capture only a small fraction of the total microbial diversity [15, 16]. The advent of 16S rRNA gene amplicon sequencing provides the first glimpse into the immense taxonomic complexity of Antarctic ecosystems, revealing the presence of distinct bacterial and archaeal lineages that are often endemic to Antarctic environments [12, 14]. Recent studies based on amplicon and metagenomic approaches have further expanded our understanding of microbial diversity and biogeographic patterns in Antarctic marine ecosystems, highlighting strong habitat-specific community structures and environmental filtering effects [12, 17–19]. However, such marker-based approaches provide limited insights into the metabolic potential, ecological roles and evolutionary histories of these micro-organisms [20]. Nevertheless, Antarctic deep-sea environments are the key players in biogeochemical processes, yet the genomic diversity and functional potential of the micro-organisms residing in these ecosystems remain inadequately characterized due to limited sampling and technical constraints [13, 21]. Many dominant taxa, particularly within *Chloroflexi*, *Planctomycetes* and *Thaumarchaeota*, remain uncultured, leaving their metabolic roles largely speculative [12, 22, 23]. Recent advances in metagenomics, single-cell genomics and metagenome-assembled genome (MAG) reconstruction have transformed microbial ecology by enabling genome-level analyses of uncultured lineages [24, 25]. These approaches reveal novel Antarctic microbes with unique cold-adaptive traits, including psychrophilic enzymes, specialized lipid biosynthesis and cold-active carbon fixation pathways [7, 13, 21, 26].

Beyond their ecological relevance, Antarctic microbes harbour significant biotechnological potential. Their cold-adaptive enzymes and diverse secondary metabolite biosynthetic gene clusters (BGCs) represent promising sources for novel biomolecules [7, 27–29]. Yet, the diversity, distribution and ecological drivers of these genomic traits remain largely unexplored, highlighting the urgent need for integrative genomic frameworks to uncover the hidden metabolic landscape of Antarctic deep-sea microbiomes [28, 29]. Furthermore, there is currently a lack of a comprehensive and curated genomic database specifically targeting Antarctic deep-sea micro-organisms, which limits our understanding of the microbial 'dark matter' and its role in polar biogeochemical cycles.

Thus, to address these gaps, we focused on Antarctic deep-sea marine environments spanning water depths of ~300–3500 m, covering a broad bathymetric gradient from continental margin waters to deep-ocean environments. Microbial communities inhabiting these environments play essential roles in organic matter transformation and biogeochemical cycling. In this study, we integrated the metagenomic sequencing data of deep-sea sediment from Prydz Bay with the publicly available Antarctic marine metagenomic datasets to establish a comprehensive genome-resolved framework for Antarctic microbial ecology. The integrated dataset comprised 25 metagenomic samples from both seawater and sediment habitats, enabling systematic investigation of microbial taxonomic composition, metabolic potential and ecological interaction networks. Collectively, this study provides a genome-resolved perspective on the structure, functional potential and ecological adaptation of Antarctic marine microbiota across different deep-sea environments. These integrated genomic insights advance our understanding of microbial organization and adaptation in extreme Antarctic ecosystems and provide a foundation for future ecological, evolutionary and biotechnological investigations.

## Methods

### Sampling and DNA extraction

Seawater and surface sediment samples used in this study consisted of 3 newly collected Antarctic surface sediment samples from Prydz Bay and 22 publicly available metagenomic datasets from Antarctic Ocean seawater and sediments. The sediment samples were collected using stainless-steel box corers and frozen after onboard processing. Genomic DNA was extracted from seawater filters and sediment aliquots using the TIANamp Genomic DNA Kit (Tiangen Biotech, Beijing, China) following the manufacturer’s protocol, ensuring high-quality DNA suitable for metagenomic sequencing.

### Metagenomic sequencing, assembly and taxonomic profiling

Metagenomic libraries were constructed using Illumina TruSeq kits and sequenced on the Illumina NovaSeq 6000 platform to generate 150 bp paired-end reads. Low-quality reads and adapter sequences were removed with Trimmomatic (SLIDINGWINDOW:4 : 20; MINLEN:50), and high-quality reads were assembled into contigs using MEGAHIT (v1.1.3) with metagenome-optimized parameters. The contig coverage profiles were calculated by mapping reads to assemblies with Bowtie2 (v2.4.5) and processing resulting SAM/BAM files using SAMtools (v1.15) (https://github.com/samtools/samtools). Taxonomic classification of the microbiome was performed using Kraken2 (v2.0.9-beta) (https://github.com/DerrickWood/kraken2) database including RefSeq bacterial, archaeal and genomes, and species abundance was estimated using Bracken (v2.6). Relative abundance profiles were visualized through stacked barplots and heatmaps using the R package ggplot2 [30], and the top 15 taxa were selected based on their average relative abundance across all samples for comparative community analysis across seawater and sediment samples. Before assembly, low-quality bases (Phred score <20) and residual Illumina adapter contaminations were excluded using fastp (v0.19.4) and Cutadapt (v1.18), respectively, and reads identified as potential non-target contamination by BMTagger (v1.1.0) were filtered out. The clean reads of each metagenome were assembled independently using MEGAHIT (v1.1.3). Across all metagenomic samples, sequencing generated an average of 86.28 million raw paired-end reads per sample (~ 37.58–135.57 million reads). After quality filtering and adapter removal, an average of 72.00 million high-quality reads per sample were retained, corresponding to 95.15% of the raw data. The sequencing depth was sufficient to support downstream assembly and genome reconstruction, as reflected by the recovery of 186 medium-quality MAGs from the combined datasets. All publicly available datasets were reprocessed using a unified bioinformatics workflow, including consistent quality control, assembly and annotation procedures, to ensure comparability across samples despite differences in original sequencing strategies.

### Genome binning, MAG refinement and quality assessment

MAGs were recovered from assembled contigs using the MetaWRAP pipeline (v1.3.2) with default parameters. The pipeline integrated three binning algorithms including MaxBin2 (v2.2.7), MetaBAT2 (v2.12.1) and CONCOCT (v1.1.0). Then, the completeness and contamination of each MAG were evaluated using CheckM v1.0.12. Afterwards, the MAGs with >50% completeness and *<*10% contamination were retained as medium-sized genomes. The MAGs were combined and dereplicated using dRep v2.6.2 with the following options: ‘-pa 0.9 -sa 1 -nc 0.30 cm larger’. Taxonomic classification of MAGs was performed using GTDB-Tk (v2.1.1) (https://gtdb.ecogenomic.org/) with default parameters [31].

### Functional gene analysis and phylogenetic tree construction

Gene prediction of the metagenomic contigs from each sample was performed using Prodigal (v2.6.3) with the parameter ‘-p meta’. The predicted genes were filtered to remove genes shorter than 100 bp. A non-redundant gene catalogue was constructed from the predicted genes by MMseqs2 (v13.45111) with the parameter ‘easy-cluster -min-seq-id 0.95 c 0.9’ to cluster the genes with the criteria of identity ≥95% and overlap ≥90%. The non-redundant gene catalogue was annotated with KofamKOALA v1.3.0 (https://www.genome.jp/tools/kofamkoala/) to assign KEGG Orthology. The standalone run_dbCAN2 (v2.0.11) (http://cys.bios.niu.edu/dbCAN2) was used to detect the presence of carbohydrate-active enzymes (CAZymes). For the MAGs, mass screening for virulence factors using the Virulence Factor Database (VFDB) (https://www.mgc.ac.cn/VFs/) was performed by using ABRicate (v1.0.1) (https://github.com/tseemann/abricate). The phylogenomic tree was reconstructed using PhyloPhlAn (v3.0.60). The tree was based on 400 universal marker genes defined in PhyloPhlAn and built using the following parameters: ‘-diversity high -fast -remove_fragmentary_entries -subsample fivehundred -min_num_markers 50’. The phylogenetic tree was visualized using iTOL (v6.1.1) [32]. BGCs were predicted by antiSMASH v7.0, revealing nonribosomal peptide synthetase (NRPS), polyketide synthase (PKS), terpene and siderophore potential across taxa. BioMGCore [33] was then used to summarize gene presence, copy number and distribution among dominant genera, following the annotation pipeline of Feng *et al*. [34]. Metagenome-derived antimicrobial peptide (AMP) candidates were annotated and compared against the AMPSphere database (v2024) (https://ampsphere.big-data-biology.org/), which was constructed based on global microbiome-derived AMP discovery using machine learning [35]. To improve robustness, predicted genes were first filtered based on sequence length and quality during gene catalogue construction, and redundancy was reduced through clustering into a non-redundant gene set. AMP annotations were assigned based on sequence similarity to curated entries in the AMPSphere database, and only database-supported matches were retained for downstream analysis. These predicted AMP candidates should be interpreted as putative annotations derived from computational inference. Plasmid sequences were predicted from metagenomic assemblies using PlasFlow (v1.1) (https://github.com/smaegol/PlasFlow). Prophage regions were identified using PHASTER (v2016) (https://www.wishartlab.com/) with default parameters.

### Abundance profiling, co-occurrence network and topological analyses

Taxonomic abundance matrices generated using Kraken2 and Bracken were used to infer microbial co-occurrence networks for seawater and sediment microbiomes. Prior to network construction, taxonomic abundance data were normalized using total-sum scaling, whereby counts were converted to relative abundances with equal total sums across samples. These output tables function similarly to count matrices commonly used in amplicon sequence variant (ASV)-based analyses for constructing co-occurrence networks. Network construction and topological property analyses were conducted using the R package igraph (v1.5.1) following the workflow described in reference [36]. Briefly, pairwise Spearman correlations were calculated among taxonomic abundance profiles derived from Kraken2 and Bracken to infer co-occurrence relationships. Correlation analyses were performed on normalized abundance data and interpreted with consideration of the compositional nature of the data. Significant correlations (|*ρ*| > 0.6, false discovery rate<0.05) were retained as network edges, where positive and negative correlations represented potential co-presence and exclusion interactions, respectively. The resulting adjacency matrices were imported into igraph (v1.5.1) for network construction and structural computation, while igraph (v1.5.1) and ggClusterNet (v0.2.6) were employed for visualization and modular layout optimization.

To assess network organization and robustness, topological indices including node degree, average path length, network diameter, clustering coefficient, density, modularity (Louvain, Walktrap and Fastgreedy), edge connectivity, graph efficiency and vulnerability were quantified. Keystone taxa were identified based on network topological features, including degree and betweenness centrality, which are commonly used to identify highly connected and potentially influential taxa within microbial co-occurrence networks. For network annotation, the top 20 genera ranked by node degree, along with the most abundant phyla represented within the networks, were highlighted to facilitate ecological interpretation and comparative evaluation of seawater and sediment interaction patterns.

## Results and discussion

### Reconstruction of MAGs from Antarctic deep-sea sequencing data

Sampling locations are shown in Fig. 1(a) and Table 1. A total of 9 Antarctic seawater and 16 Antarctic sediment samples (3 collected from Prydz Bay and 13 obtained from public datasets) were included, all from depths exceeding 300 m. Using the workflow illustrated in Fig. 1(b), we reconstructed MAGs and established an integrated gene catalogue. From these metagenomic datasets, a total of 186 dereplicated MAGs (>50% completeness, <10% contamination) had been recovered, including 90 MAGs from sediments and 96 MAGs from seawater (Table S1, available in the online Supplementary Material). These comprised 175 bacterial and 11 archaeal genomes spanning 19 phyla, substantially expanding the currently available genomic resources when compared with previous studies, for example, 61 MAGs reported by Garber *et al*. [13], providing a more comprehensive framework for ecological interpretation. Despite increasing efforts to characterize Antarctic marine microbiomes, systematic genome-resolved investigations of deep-sea communities remain limited [26]. Therefore, the recovered MAGs provide complementary insights into the genomic diversity of Antarctic micro-organisms inhabiting deep-sea environments. Therefore, the recovered MAGs provide complementary insights into the genomic diversity of Antarctic micro-organisms inhabiting less-explored deep-sea environments.

**Fig. 1.**
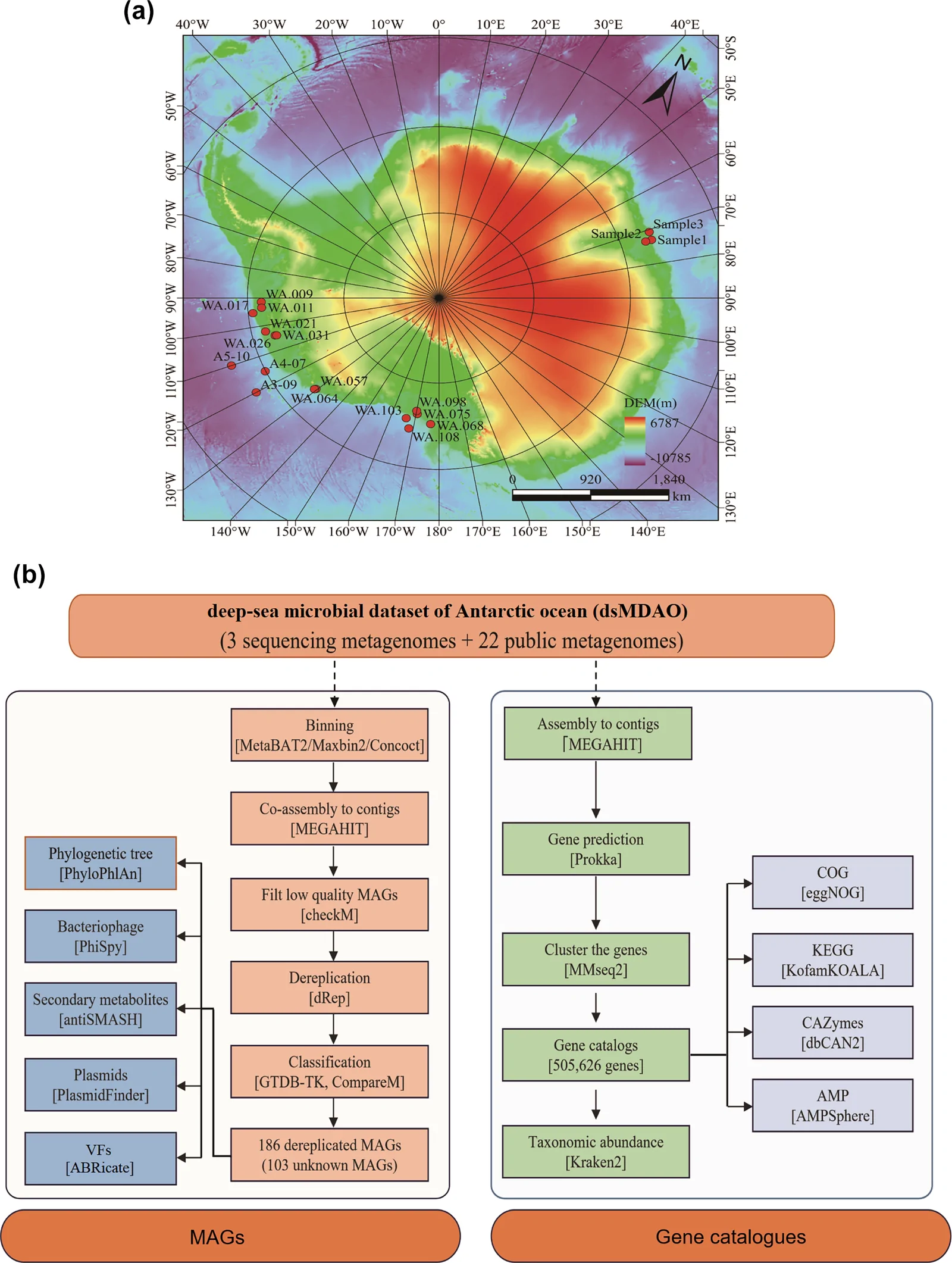
Antarctic sampling site distribution and metagenomic analysis workflow. (**a**) Map of the Antarctica Ocean, showing the geographic locations from which the metagenomic samples (*n*=25) were derived. Due to spatial proximity, some sampling points may overlap on the map. Among these, 3 samples were newly collected and sequenced in this study, while the remaining 22 samples were retrieved from publicly available databases. (**b**) Flowchart of the bioinformatic workflow applied for assembly, binning, annotation and analysis of metagenomic data and gene catalogues. Metagenomic sequencing data from 25 Antarctic deep-sea samples (three newly sequenced and 22 publicly available datasets) were processed independently. Reads from each sample were assembled into contigs using MEGAHIT, followed by genome binning with MetaWRAP. The resulting MAGs (>50% completeness and <10% contamination) were dereplicated and classified. Downstream analyses included phylogenomic reconstruction, secondary metabolite prediction, virulence factor identification, antimicrobial resistance gene annotation, plasmid prediction using PlasFlow and bacteriophage detection using PhiSpy. Gene catalogues were functionally annotated using KEGG (KofamKOALA), CAZymes (dbCAN2), eggNOG (COG/GO annotation) and AMP identification (AMPSphere). The workflow arrows have been adjusted to accurately represent the data processing steps.

**Table 1.** Background of the deep-sea public data (*n*=22) and the newly sequenced metagenomic data (*n*=3)

Type	Sample ID	Location	BioProject/SRA	Longitude (E)	Latitude (S)	Depth (m)
Antarctic-sediment	Sample1 2700 m	Prydz Bay1 (this study)	PRJNA1267735	72.99°	−66.85°	2,700
Antarctic-sediment	Sample2 2700 m	Prydz Bay2 (this study)	PRJNA1267735	73°	−67.49°	2,700
Antarctic-sediment	Sample3 2700 m	Prydz Bay3 (this study)	PRJNA1267735	70.64°	−66.8°	2,700
Antarctic-deep-sea	A3-09-300m-34An	Antarctic deep sea [17]	SRR10912860; SRR10912861	−120°	−68.01°	300
Antarctic-deep-sea	A4-07-300m-34An	Antarctic deep sea [17]	SRR10912878; SRR10912879	−115.24°	−69.94°	300
Antarctic-deep-sea	A5-10-300m-34An	Antarctic deep sea [17]	SRR10912869; SRR10912870	−110°	−67.02°	300
Antarctic-deep-sea	A3-09-1000m-34An	Antarctic deep sea [17]	SRR10912858; SRR10912859	−120°	−68.01°	1,000
Antarctic-deep-sea	A4-07-1000m-34An	Antarctic deep sea [17]	SRR10912876; SRR10912877	−115.24°	−69.94°	1,000
Antarctic-deep-sea	A5-10-1000m-34An	Antarctic deep sea [17]	SRR10912867; SRR10912868	−110°	−67.02°	1,000
Antarctic-deep-sea	A4-07-3000m-34An	Antarctic deep sea [17]	SRR10912874; SRR10912875	−115.24°	−69.94°	3,000
Antarctic-deep-sea	A3-09-3500m-34An	Antarctic deep sea [17]	SRR10912856; SRR10912857	−120°	−68.01°	3,500
Antarctic-deep-sea	A5-10-3500m-34An	Antarctic deep sea [17]	SRR10912865; SRR10912866	−110°	−67.02°	3,500
Antarctic-sediment	WA.009	Bellingshausen Sea 1 [16]	SRR10156275	−91.43°	−71.42°	412
Antarctic-sediment	WA.011	Bellingshausen Sea 2 [16]	SRR10156274	−93.48°	−71.42°	672
Antarctic-sediment	WA.017	Bellingshausen Sea 3 [16]	SRR10156270	−95.2°	−70.48°	476
Antarctic-sediment	WA.021	Amundsen Sea 1 [16]	SRR10156269	−102.16°	−71.45°	471
Antarctic-sediment	WA.026	Amundsen Sea 2 [16]	SRR10156268	−104.3°	−72.3°	597
Antarctic-sediment	WA.031	Amundsen Sea 3 [16]	SRR10156267	−104.36°	−72.46°	572
Antarctic-sediment	WA.057	Wrights Gulf 1 [16]	SRR10156266	−129.56°	−73.3°	510
Antarctic-sediment	WA.064	Wrights Gulf 2 [16]	SRR10156265	−129.27°	−73.16°	478
Antarctic-sediment	WA.068	Ross Sea 1 [16]	SRR10156264	−176.59°	−75.2°	567
Antarctic-sediment	WA.075	Ross Sea 2 [16]	SRR10156263	−170.51°	−76.2°	531
Antarctic-sediment	WA.098	Near Ross Shelf 1 [16]	SRR10156273	−170°	−76.55°	765
Antarctic-sediment	WA.103	Near Ross Shelf 2 [16]	SRR10156272	−166.3°	−75.5°	552
Antarctic-sediment	WA.108	Near Ross Shelf 3 [16]	SRR10156271	−168.30°	−74.4°	528

Based on the taxonomic annotation of the recovered MAGs, *Proteobacteria* was the dominant phylum (*n*=102), followed by *Bacteroidota* (*n*=27), *Actinobacteriota* (*n*=7), *Chloroflexota* (*n*=7), *Thermoplasmatota* (*n*=6), *Verrucomicrobiot*a (*n*=5), *Thermoproteota* (*n*=5), *Nitrospinota* (*n*=5), *Nitrospirota* (*n*=4) and *Marinisomatota* (*n*=4), with several minor phyla also detected. At the genus level, seawater-derived MAGs were dominated by metabolically versatile taxa such as *Alcanivorax*, *Marinobacter*, *Sphingomonas*, *Pseudomonas* and *Altererythrobacter*, whereas sediment-associated communities were enriched in *Anaerolinea*, *Nitrosopumilus* and uncultured *Chloroflexi* and *Planctomycetota*-related lineages (Table S1). This clear taxonomic partitioning between seawater and sediment habitats highlights strong environmental filtering driven by depth, redox conditions and substrate availability [37, 38], as well as pronounced spatial variation in Antarctic benthic microbial communities [39] and geochemically structured microbial assemblages in Antarctic sediments [40]. Specifically, seawater communities appear dominated by opportunistic and flexible taxa adapted to fluctuating nutrient regimes and oxidative stress, while sediment communities harbour more phylogenetically diverse and specialized lineages consistent with low-energy, microoxic and organic-rich conditions. These patterns are consistent with observations from other marine systems, where environmental gradients shape microbial community assembly and functional differentiation [37, 38, 41, 42]. Notably, a substantial proportion of MAGs belonged to uncultured or poorly characterized lineages, underscoring the prevalence of ‘microbial dark matter’ in Antarctic ecosystems [14, 25], which are likely adapted to oligotrophic and cold environments through genome streamlining and alternative metabolic strategies such as mixotrophy [7, 43, 44].

### Diversity of microbial community composition in the deep sea of Antarctica

To characterize differences in microbial community composition among Antarctic deep-sea habitats, taxonomic profiles were generated for the 25 metagenomic samples and compared at both the phylum and genus levels. Bray–Curtis principal coordinate analysis (PCoA) revealed a clear separation among Prydz Bay sediments, other sediment samples and seawater samples at the phylum level, with the first two principal coordinates explaining 49.0 and 10.7% of the total variation, respectively (Figs 2a and S1). Permutational multivariate analysis of variance (PERMANOVA) further confirmed significant differences in community composition among the three groups (*R*² = 0.676, *P*<0.001). A similar pattern was observed at the genus level, where PCoA1 and PCoA2 explained 37.0 and 14.9% of the variation, respectively, and the three habitat groups remained significantly differentiated (PERMANOVA, *R*² = 0.571, *P*<0.001; Fig. 2b).

**Fig. 2.**
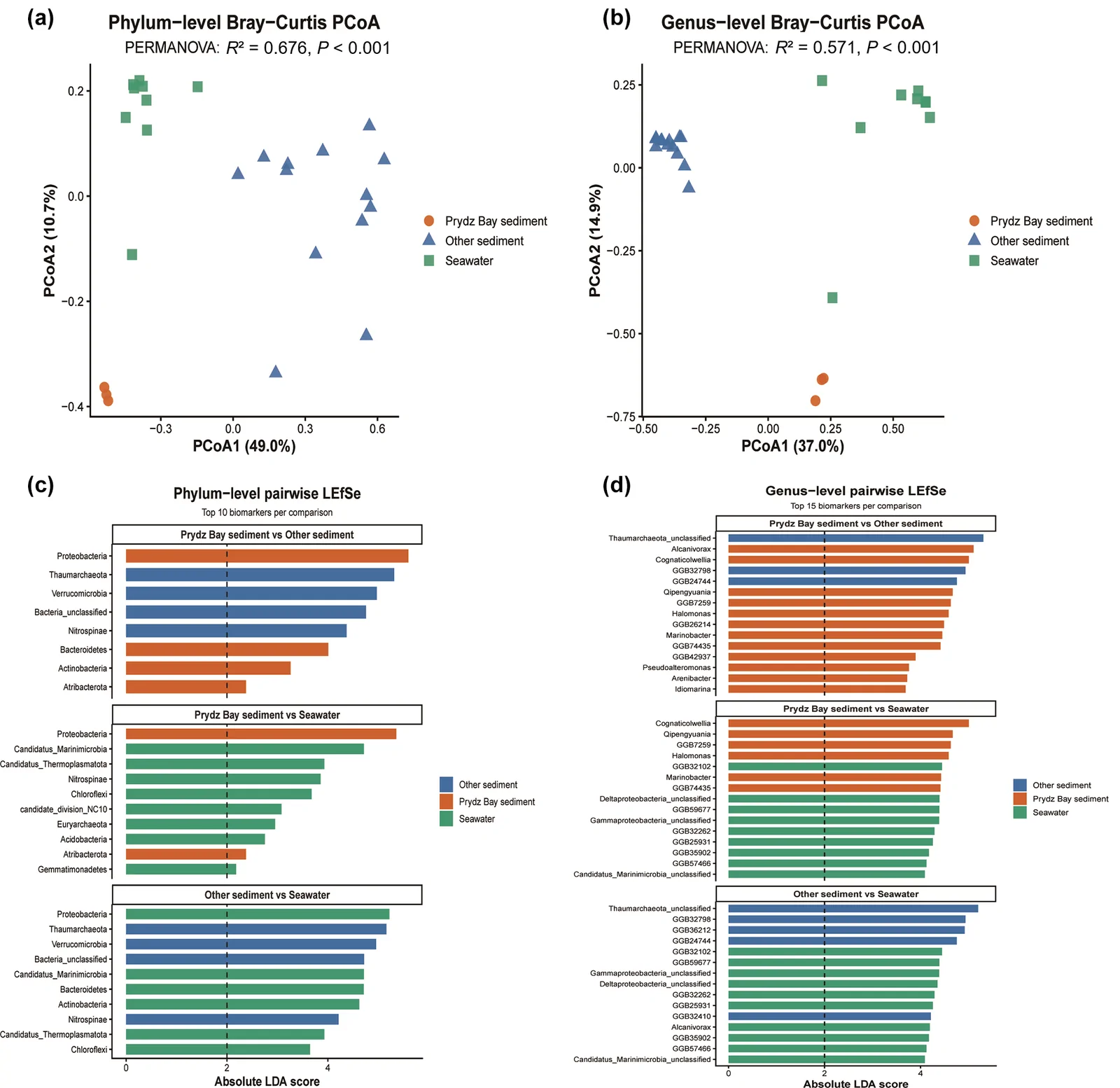
Differences in microbial community composition and habitat-associated taxa among Antarctic deep-sea samples. (**a**) Bray–Curtis PCoA of microbial community composition at the phylum level. PCoA1 and PCoA2 explained 49.0 and 10.7% of the total variation, respectively, and PERMANOVA confirmed significant differences among Prydz Bay sediments, other sediment samples and seawater samples (*R*² = 0.676, *P*<0.001). (**b**) Bray–Curtis PCoA at the genus level, with PCoA1 and PCoA2 explaining 37.0 and 14.9% of the variation, respectively (PERMANOVA, *R*² = 0.571, *P*<0.001). (**c**) Pairwise linear discriminant analysis effect size (LEfSe) analysis at the phylum level showing the top ten discriminative taxa for comparisons between Prydz Bay sediments and other sediments, Prydz Bay sediments and seawater and other sediments and seawater. (**d**) Pairwise LEfSe analysis at the genus level showing the top 15 discriminative taxa for each comparison. Bar colours indicate the habitat group with which each taxon was preferentially associated, and bar length represents the absolute linear discriminant analysis (LDA) score.

Taxonomic abundance profiles further demonstrated marked habitat-specific differences (Table S2). Prydz Bay sediments were strongly dominated by *Proteobacteria*, which accounted for ~96% of the microbial community on average, whereas *Bacteroidetes* represented the second most abundant phylum. In contrast, other sediment samples were characterized by relatively high abundances of *Thaumarchaeota*, *Verrucomicrobia*, *Proteobacteria* and unclassified bacterial lineages, while seawater communities were mainly composed of *Proteobacteria*, *Thaumarchaeota*, *Bacteroidetes*, Candidatus *Marinimicrobia* and *Actinobacteria*. These compositional differences were further supported by pairwise LEfSe analysis (Fig. 2c). Proteobacteria was strongly associated with Prydz Bay sediments, whereas *Thaumarchaeota*, *Verrucomicrobia*, unclassified Bacteria and *Nitrospinae* were characteristic of other sediment samples. Several lineages, including Candidatus *Marinimicrobia*, Candidatus *Thermoplasmatota*, *Chloroflexi* and *Actinobacteria*, were preferentially associated with seawater communities.

At the genus level, Prydz Bay sediments were characterized by a distinct assemblage dominated by *Alcanivorax* and *Cognaticolwellia*, together with *Qipengyuania*, GGB7259, *Halomonas*, GGB26214, *Marinobacter* and GGB74435 (Table S1). In contrast, other sediment samples were strongly associated with *Thaumarchaeota*_unclassified and several uncultured GGB lineages, including GGB32798, GGB36212 and GGB24744. Seawater samples contained a broader set of unclassified or genome-based genera, including GGB32102, GGB59677, GGB32262, GGB25931 and Candidatus_*Marinimicrobia*_unclassified. Pairwise LEfSe analysis confirmed these habitat-specific genus-level signatures (Fig. 2d), highlighting pronounced taxonomic differentiation among Prydz Bay sediments, other Antarctic deep-sea sediments and seawater communities.

By integrating genome-resolved MAGs with read-based taxonomic profiles, we obtained complementary views of microbial diversity across Antarctic deep-sea habitats. GTDB-based classification of the 186 recovered MAGs (Table S1) showed that the genome-resolved dataset was predominantly bacterial, with *Proteobacteria* representing the largest fraction of reconstructed genomes and being particularly well represented in Prydz Bay sediments. In contrast, archaeal MAGs were recovered from other sediment and seawater samples, consistent with the stronger representation of archaeal lineages in these habitats in the read-based profiles. Importantly, the distribution of recovered MAGs should not be interpreted as a direct measure of taxon abundance, because successful genome reconstruction depends on sequencing coverage, assembly efficiency and binning quality. Reads-based profiling therefore complements genome reconstruction by capturing community signals from lineages whose genomes were not successfully recovered as MAGs, including archaeal groups and numerous low-abundance or poorly characterized taxa. This complementarity is consistent with recognized methodological differences between assembly- and read-based approaches [25, 45] and provides a more comprehensive representation of Antarctic deep-sea microbial communities.

Ecologically, the strong habitat-associated differentiation observed in both analyses suggests that distinct environmental conditions structure microbial communities across Antarctic deep-sea habitats. The overwhelming predominance of *Proteobacteria* in Prydz Bay sediments, together with *Bacteroidetes* as the second most abundant phylum in the read-based profiles, is consistent with a community adapted to relatively greater availability of organic substrates. In contrast, the enrichment of *Thaumarchaeota* in other sediment samples is consistent with the ecological importance of ammonia oxidation under energy-limited conditions [23], while *Verrucomicrobia* and *Nitrospinae* further distinguished these sediment communities. Seawater communities formed a taxonomically distinct assemblage characterized by *Proteobacteria*, *Thaumarchaeota*, *Bacteroidetes*, Candidatus *Marinimicrobia* and *Actinobacteria*, potentially reflecting selection under oligotrophic conditions and water-column mixing. Notably, the frequent occurrence of unclassified GGB lineages in the read-based profiles and UBA-designated lineages among the reconstructed MAGs highlights the substantial poorly characterized component of Antarctic microbial diversity [14, 25]. Previous genomic studies of uncultured microbial lineages have identified genome streamlining and flexible, including potentially mixotrophic, metabolic strategies as adaptations to cold and nutrient-poor environments [7, 43, 44]. Such traits could facilitate persistence under polar deep-sea conditions and contribute to sustained biogeochemical transformations despite low rates of microbial activity [13, 26]. These habitat-associated patterns are consistent with previous studies across Antarctic marine environments showing that physicochemical gradients, seasonal ice dynamics and water-mass characteristics can strongly influence microbial community assembly and biogeographic distribution [8, 17, 46, 47]. The coexistence of widely distributed marine taxa and numerous poorly characterized lineages further suggests that Southern Ocean microbial biogeography reflects the combined influence of dispersal and local environmental selection. By linking read-based community profiles with reconstructed MAGs, our study therefore extends community-level patterns of habitat differentiation into a genome-resolved framework.

### Functional traits of Antarctic microbial genomes

Genome-resolved analyses revealed diverse and ecologically specialized functional traits across the 186 Antarctic MAGs (Fig. 3, Tables S1 and S3-S8). These features encompass extensive secondary-metabolite biosynthetic capacities, variable virulence-associated gene repertoires and distinct metabolic adaptations, reflecting the genomic strategies that sustain microbial survival and interaction networks under extreme polar conditions. To further resolve evolutionary relationships and functional potential, a phylogenomic tree based on the 175 MAGs was constructed and integrated with multilayered annotations, including taxonomy, secondary metabolite BGCs, virulence factors and CAZymes (Fig. 3), providing genome-resolved insights into the relationship between phylogeny and ecological function in Antarctic deep-sea microbiomes. The secondary metabolite BGCs were widespread among the recovered genomes, occurring in both seawater- and sediment-derived taxa (Fig. 3; Tables S1, S3 and S6). BGC richness was particularly high within *Proteobacteria* and *Bacteroidota*, emphasizing their metabolic versatility and ecological prominence in Antarctic marine systems. Similar biosynthetic potential has also been reported in Antarctic coastal sediment-associated microbial communities, where *Proteobacteria* and *Bacteroidota* contribute substantially to BGC diversity [29].

**Fig. 3.**
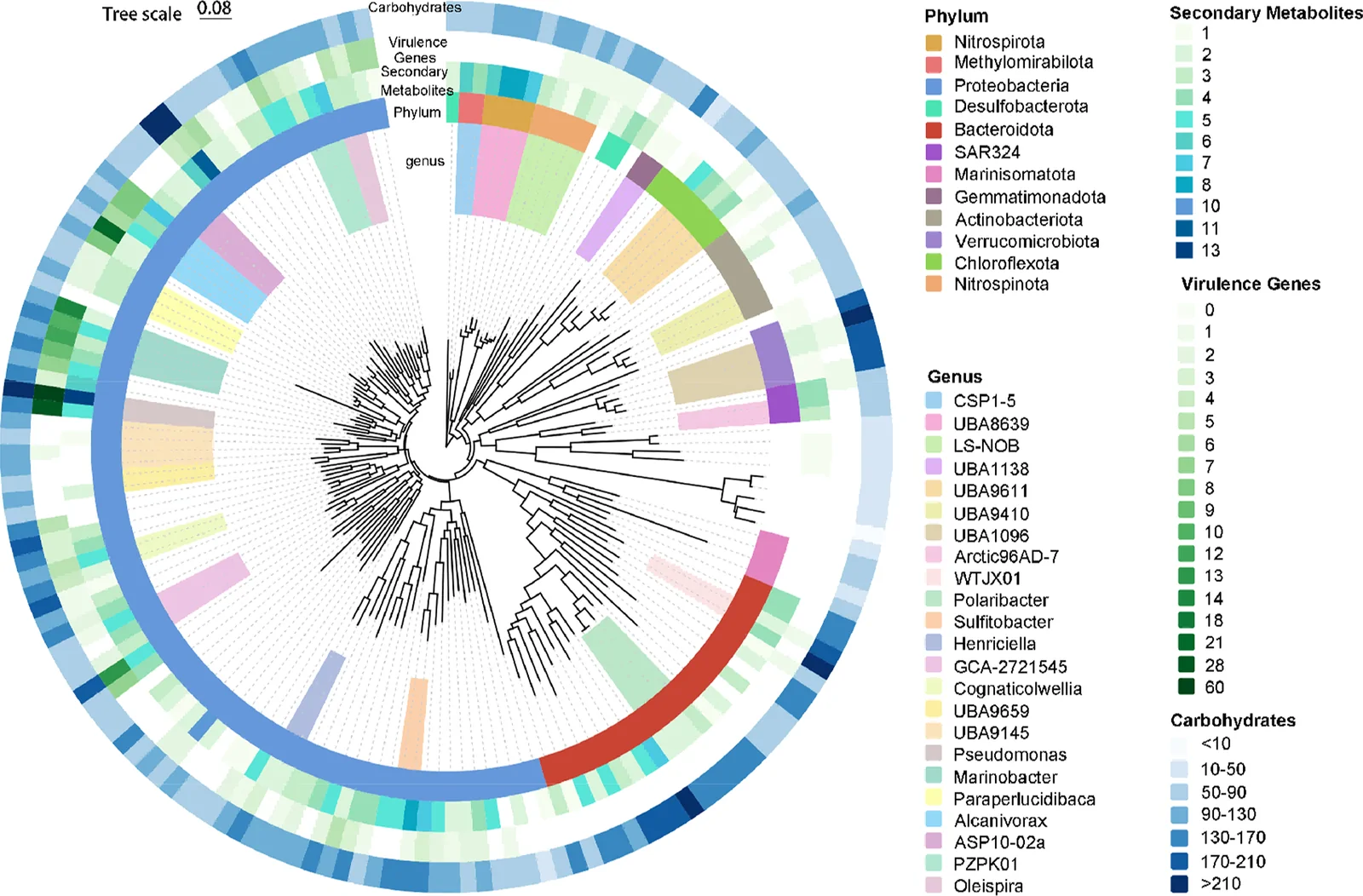
Phylogenetic tree and multi-layered functional summary of 175 bacterial MAGs recovered from Antarctic deep-sea sediments and seawater. The circular phylogenomic tree was constructed from 175 bacterial MAGs recovered from 25 Antarctic deep-sea metagenomes, including 16 sediment and 9 seawater samples. The inner annotation ring represents genus-level taxonomic assignments, highlighting both characterized genera such as *Marinobacter*, *Antarcticibacter* and *Oleispira* and uncultured or genome-based lineages such as UBA8639, GCA-2721545 and CSP1-5. The phylum-level ring represents 12 major bacterial phyla, including *Proteobacteria*, *Bacteroidota*, *Actinobacteriota*, *Desulfobacterota*, SAR324 and *Chloroflexota*. The three outer functional layers quantitatively summarize different genomic features for each MAG: the number of predicted secondary-metabolite BGCs, the number of distinct VFDB-annotated virulence-associated gene types and the total number of dbCAN CAZyme annotations (GH, glycoside hydrolase; GT, glycosyl transferase; CBM, carbohydrate-binding module; CE, carbohydrate esterase; AA, auxiliary activity; and PL, polysaccharide lyase), respectively. Colour intensity in these three layers represents only the numerical count of the corresponding genomic feature for each MAG and does not represent specific functional identities. Detailed BGC types and closest known clusters, virulence-associated gene identities and class-resolved CAZyme annotations are provided in Tables S6–S8, respectively.

At the genus level, UBA8639 (29 clusters), *Alcanivorax* (*n*=27), UBA9611 (*n*=17), PZPK01 (*n*=15), CSP1-5 (*n*=14), *Marinobacter* (*n*=14), *Pseudomonas* (*n*=13), *Sulfitobacter* (*n*=13) and GCA-2721545 (*n*=12) were the most biosynthetically active lineages. These BGCs included diverse cluster types such as NRPS, PKS, terpenes and aryl-polyenes, which are widely associated with the biosynthesis of structurally and functionally diverse secondary metabolites [28, 29].

Habitat comparison between seawater and sediment revealed that seawater-abundant genera (*Alcanivorax*, *Marinobacter* and *Pseudomonas*) based on taxonomic abundance profiles derived from MAGs (Table S1), typically harboured relatively higher numbers of BGCs per genome, whereas sediment-associated taxa such as *Henriciella* and *Sulfitobacter* showed variable BGC distributions across genomes. The observed patterns suggest potential differences in biosynthetic features between seawater- and sediment-associated micro-organisms; however, these trends should be interpreted with caution due to limitations in data integration across datasets. Archaeal genomes contained relatively few recognizable BGCs, although direct comparison was limited by their absence in the phylogenomic tree.

The distribution of virulence-associated genes exhibited phylogenetic and ecological variation (Fig. 3; Tables S1, S4 and S7). Some genera, such as *Pseudomonas*, displayed relatively higher numbers of virulence-associated genes, while others showed more variable and generally lower counts across genomes. These genes predominantly encoded components of secretion systems (types II, III and VI), adhesion and motility structures, toxin–antitoxin modules, siderophore-mediated iron acquisition systems and oxidative stress-response enzymes. Moderate virulence gene counts (10–20 per genome) were identified in *Pseudomonas*, *Marinobacter* and *Sphingomonas*, mainly associated with interspecies competition, stress resistance and biofilm formation. Importantly, these annotations were obtained by sequence comparison against VFDB, which is primarily curated from experimentally characterized virulence factors of bacterial pathogens; therefore, their detection in environmental MAGs should not be interpreted as direct evidence of pathogenicity [48].

In contrast, lineages belonging to *Nitrospinota*, *Chloroflexota* and *Actinobacteriota* possessed few or no virulence-related genes, indicating more restricted repertoires of annotated virulence-associated functions in these lineages. The widespread yet non-pathogenic nature of these virulence determinants suggests that they may primarily contribute to ecological defence, niche competition and environmental adaptation rather than directly indicating pathogenicity [49]. The distribution and diversity of CAZyme families across Antarctic MAGs were strongly shaped by both taxonomy and habitat (Fig. 3; Tables S1, S5 and S8). Members of the *Bacteroidota* particularly those belonging to the order *Flavobacteriales* displayed the highest CAZymes richness and diversity, especially in glycoside hydrolases (GHs) and polysaccharide lyases, underscoring their role as primary degraders of complex polysaccharides. This finding is consistent with previous genomic studies demonstrating extensive carbohydrate-active repertoires and polysaccharide-utilization potential in marine *Bacteroidota* and *Flavobacteriales* [50, 51]. In contrast, *Proteobacteria* showed relatively lower CAZyme counts on a per-genome basis, although several *Gammaproteobacteria* (*Cellvibrionaceae*, *Marinobacter* and *Oleispira*) still possessed extensive carbohydrate-active repertoires. Other bacterial phyla such as *Actinobacteriota*, *Chloroflexota* and *Gemmatimonadota*, along with most Archaea, exhibited minimal or specialized CAZyme inventories, typically restricted to GH or carbohydrate esterase families linked to refractory organic matter turnover. Thus, the present genome-resolved analysis extends previous observations of taxon-dependent polysaccharide metabolic potential by showing substantial variation in CAZyme repertoires among Antarctic deep-sea microbial lineages [50, 51].

Environmentally, MAGs recovered from sediment habitats notably from Prydz Bay and the Ross Sea harboured markedly higher CAZyme gene densities and a broader enzymatic spectrum than those from the deep-sea water column (Fig. 3; Tables S5 and S8). This pattern mirrored the greater heterogeneity and organic-matter complexity of the benthic environment. Sediments from Prydz Bay and the Ross Sea formed distinct ‘CAZyme hotspots’, with certain MAGs such as *Arenibacter* containing exceptionally large CAZyme inventories (>160 GHs). Conversely, in the water column, CAZyme profiles were generally reduced, although specific *Gammaproteobacteria* lineages retained notable degradative potential, suggesting functional adaptation to suspended organic substrates.

Similarly, 24,957 AMPs were identified in sediment samples compared with only 3,165 in seawater (Table S9). These sequences were identified computationally through comparison with AMPSphere [35] and should therefore be interpreted as predicted AMP candidates rather than experimentally validated AMPs. Moreover, because the sediment and seawater datasets differed in sample number (16 versus 9) and originated from multiple sequencing datasets, the raw totals should not be interpreted as normalized evidence that benthic communities possess intrinsically greater AMP abundance or stronger competitive activity. Nevertheless, the large number of predicted candidates highlights the potential value of Antarctic deep-sea metagenomes for future AMP discovery and experimental characterization [35].

Based on the provided results, all identified genomic regions across the various samples represent incomplete phage sequences (labelled as ‘incomplete’ with scores in parentheses). The regions range in length from ~3.3 to 20.5 kb. A key consistent finding is that 100% of the predicted proteins within these regions are either phage-hit or hypothetical proteins, with no bacterial proteins detected. Each region shows hits to multiple phage species (3–11 different species), indicating sequence similarity to diverse phages (Table S10). These results indicate the presence of diverse putative phage-derived genomic fragments, although their incomplete status prevents inference of intact or active prophages from the present data alone.

As shown in Table S11, this dataset comprises 1,419 plasmid fragments derived from deep-sea micro-organisms in Antarctic seawater and sediments. The fragments are taxonomically classified based on predicted host origin, with the majority belonging to *Proteobacteria* (962 fragments), followed by unclassified plasmids (*n*=411), and a small number associated with *Firmicutes* (*n*=36) and *Actinobacteria* (9 fragments). This distribution highlights the prominent role of *Proteobacteria* in hosting plasmids within this extreme polar marine environment, while the substantial proportion of unclassified elements suggests the presence of considerable novel plasmid diversity awaiting further characterization. Thus, *Proteobacteria* represented the largest taxonomically assigned group among the predicted plasmid fragments. However, because these assignments were computational predictions, they should not be interpreted as direct evidence that *Proteobacteria* serve as the predominant plasmid hosts throughout Antarctic deep-sea ecosystems. The substantial proportion of unclassified fragments further indicates that the taxonomic affiliations of many predicted mobile genetic elements remain unresolved.

### Functional profiling reveals metabolic adaptations of Antarctic deep-sea microbiota

When the functions of the Antarctic deep-sea microbiota were analysed, we identified metabolic features that are consistent with broader ecological strategies previously reported across Antarctic marine environments [26]. A total of 6,932,707 orthologues were derived from the 25 metagenomic assemblies, resulting in an Antarctic deep-sea reference gene catalogue. The KEGG enrichment analysis of the Antarctic deep-sea metagenomic data revealed distinct functional adaptations (Fig. 4a). Environmental information processing constituted the dominant category (26.3%), followed by cellular processes (24.3%) and metabolism (17.9%), reflecting genomic priorities for extreme environment sensing and homeostasis maintenance. Notably, organismal systems represented only 1.2% of annotated genes. Key functional genes exhibited iron acquisition specialization (K02014) and cold adaptation mechanisms (K03704). Metabolic signatures included lipid metabolism enzymes (K00249, K00626) and stress-response systems (K00799). Chemotaxis regulation appeared through methyl-accepting proteins (K03406) and two-component systems (K02483). The ABC transporter prevalence (K02003/K02004/K01990) and TonB-ExbBD complex (K03559/K03561/K03832) suggest sophisticated nutrient scavenging strategies under oligotrophic conditions (Fig. 4b) [52]. These genomic features are consistent with microbial strategies for persistence in cold and resource-limited Antarctic marine environments [26]. Furthermore, the metabolism of carbon, nitrogen and sulphur in micro-organisms in the deep-sea environments was analysed. The results revealed that Antarctic microbial genera harbour a wide array of functional genes associated with nitrogen and sulphur metabolism, including *napA*, *napB*, *nirB* and *nirD* for nitrate and nitrite reduction, as well as *cysD*, *cysN* and *sat* for assimilatory sulphate pathways (Fig. 4c, Table S12). The broad distribution of these genes indicates substantial metabolic flexibility in nitrogen and sulphur acquisition and transformation, which may allow micro-organisms to exploit multiple nutrient sources and maintain metabolic activity under the nutrient-limited conditions characteristic of Antarctic marine environments. Similar metabolic strategies involving nitrogen and sulphur transformations have been reported in Antarctic marine microbial communities and are considered important for persistence in cold and oligotrophic environments [13, 53]. Notably, the genera such as g_UBA8639, g_*Oleispira* and g_*Antarcticibacter* exhibited pronounced metabolic versatility (Fig. 4c, Table S12). In particular, g_UBA8639 contained up to eight MAGs encoding the adenylyl-sulphate transferase CysN (K00957), indicating a potentially central role in sulphur reduction or sulphate assimilation. Genes involved in lipid biosynthesis, including *acsL* (K01897) and *otsB* (K01087), were broadly detected in *Marinobacter*, *Oleispira* and *Antarcticibacter*, suggesting that membrane-lipid remodelling may be an important cold-adaptation strategy across these taxa (Fig. 4c, Table S9). Additionally, genes associated with the C4-dicarboxylate carbon fixation pathway such as *ppc* were identified in multiple genera, implying the use of low-energy, resource-efficient anaplerotic carbon assimilation routes to cope with the oligotrophic conditions characteristic of Antarctic marine environments (Fig. 4c, Table S12). Collectively, these findings demonstrate that Antarctic deep-sea micro-organisms possess substantial metabolic flexibility, consistent with the ecological strategies observed across Antarctic marine ecosystems, where micro-organisms mediate carbon turnover and nutrient regeneration under persistent cold and nutrient limitation [10, 47]. The functional repertoire of Antarctic microbiota reveals coordinated strategies that may facilitate survival and ecological persistence under extreme deep-sea conditions. The widespread occurrence of genes involved in nitrogen and sulphur transformations, including *napA*, *nirB* and *cysN*, indicates broad capacities for nitrate/nitrite reduction and sulphate assimilation, thereby expanding nutrient acquisition and transformation strategies under resource-limited conditions. These metabolic functions, together with sulphur-associated pathways enriched in uncultured lineages, may contribute to the metabolic versatility and biogeochemical roles of Antarctic deep-sea micro-organisms [13, 40, 54]. Concurrently, genes related to membrane remodelling and cryoprotection (*acsL*, *otsB*) highlight key adaptations for maintaining cellular integrity under low temperature and high hydrostatic pressure [55, 56]. Archaeal MAGs further contribute to these processes through energy-efficient pathways such as ammonia oxidation and carbon fixation via the 3-hydroxypropionate/4-hydroxybutyrate cycle, underscoring their importance in deep-sea nitrification and carbon fluxes [23]. The occurrence of C4-dicarboxylate fixation genes across diverse bacterial taxa further suggests the use of low-energy carbon assimilation strategies to cope with oligotrophic conditions [57].

**Fig. 4.**
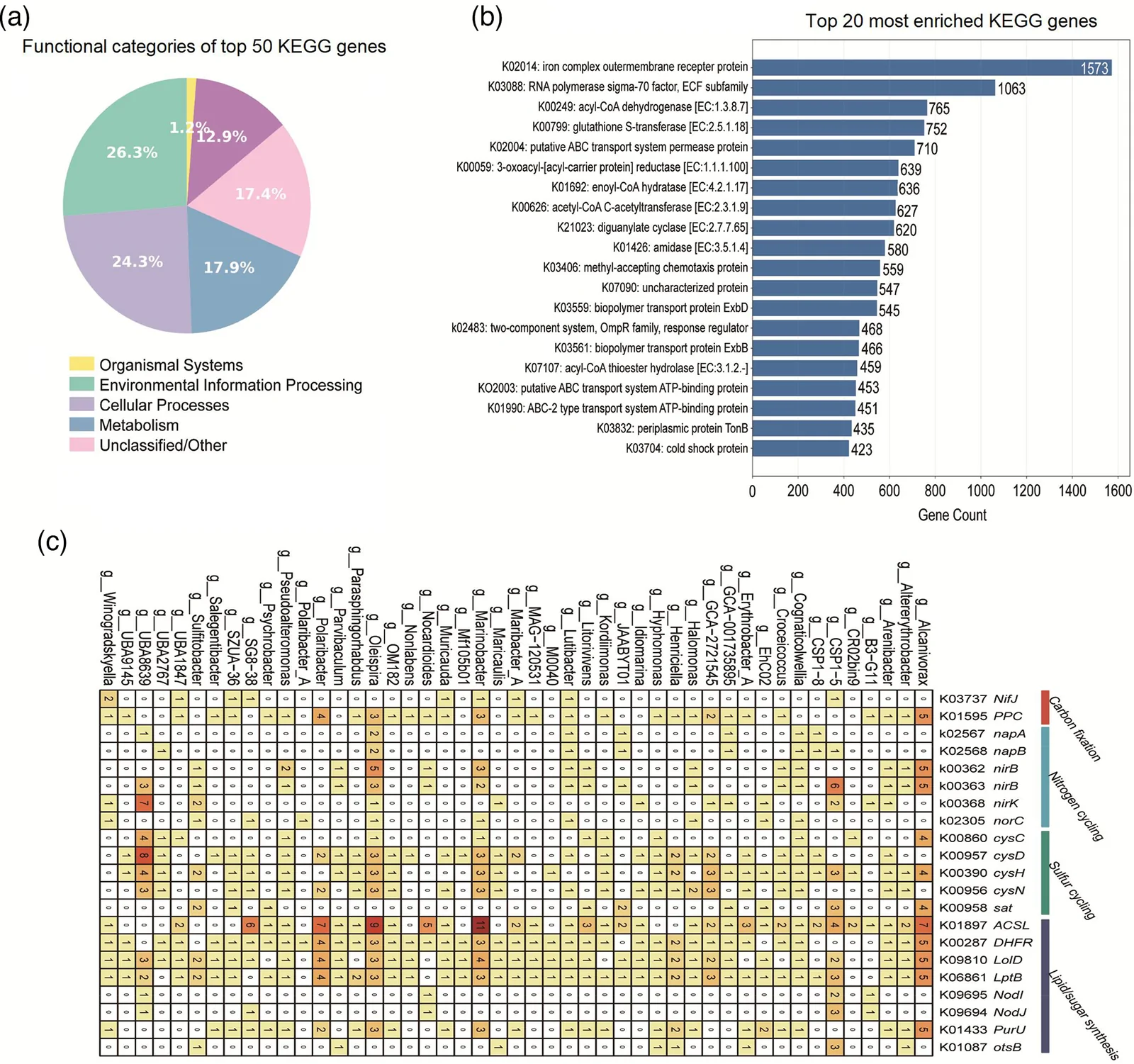
Functional gene profiles of Antarctic deep-sea microbiota. The functional categories of top 50 KEGG genes (**a**) and top 20 enriched KEGG genes (**b**) showed main microbial biological processes. (**c**) Microbial genera involved in the carbon fixation, nitrogen cycling, sulphur cycling and lipid/sugar biosynthesis process. Heatmap showing the distribution and copy numbers of key metabolic genes annotated from MAGs reconstructed from Antarctic marine sediments (*n*=16) and seawater (*n*=9). A total of 47 classified genera were examined. Functional genes related to major biogeochemical pathways including carbon fixation (*nifJ*, *PPC*), nitrogen cycling (*napA*, *napB*, *nirB* and *nirD*), sulphur cycling (*cysC*, *cysD*, *cysN* and *sat*) and lipid/sugar synthesis (*acsL*, *dhfR*, *lpxB*, *nodI*, *purU* and *otsB*) were identified through blastp annotation against the KEGG database. Colour intensity represents gene copy number within each genus. The widespread and heterogeneous distribution of these functional genes illustrates the metabolic versatility, ecological specialization and cold-environment adaptation strategies of Antarctic microbial taxa across both sediment and water column habitats.

Beyond core metabolism, Antarctic micro-organisms exhibit extensive capacities for ecological interaction and chemical mediation. The widespread distribution of secondary metabolite BGCs indicates active roles in competition and signalling under nutrient limitation [28, 29], while virulence-associated genes likely function in non-pathogenic ecological processes such as interspecies competition, iron acquisition and stress tolerance [48, 49]. In parallel, the enrichment of CAZymes, particularly in sediment-associated taxa, underscores the critical role of benthic communities in degrading complex organic matter and sustaining carbon turnover in polar ecosystems [50, 51, 58]. Consistently, KEGG functional profiles reveal enrichment in stress response, iron acquisition and transport systems, reflecting adaptive strategies for survival in energy-limited environments.

Notably, the markedly higher abundance of AMPs in sediments compared to seawater (24,957 vs. 3165 predicted AMPs, respectively) further highlights intensified microbial interactions and competitive pressures in benthic habitats, reinforcing their role as hotspots of chemical defence and ecological complexity (Table S9). From an applied perspective, these functional attributes identify Antarctic micro-organisms as a largely untapped reservoir of cold-active enzymes and bioactive compounds, including NRPS- and PKS-derived metabolites with pharmaceutical potential, as well as enzymes relevant for industrial catalysis and environmental biotechnology [7, 27, 28, 59–62]. However, the high proportion of uncultured lineages underscores the need for integrative approaches combining multi-omics and cultivation strategies to validate functional predictions and elucidate ecological roles. Collectively, these findings demonstrate that Antarctic microbiomes are shaped by tightly coupled metabolic flexibility and interaction-driven processes, supporting ecosystem stability and resilience in one of Earth’s most extreme environments.

### Co-occurrence networks unpack divergent interaction patterns in Antarctic marine habitats

Co-occurrence network analysis revealed clear structural differences between Antarctic seawater and sediment microbial communities (Fig. 5, Table S13). The sediment network was not constructed from the three Prydz Bay samples alone, but from 13 publicly available Antarctic sediment metagenomes collected from comparable bathyal depths (~412–765 m), which likely reduces large-scale environmental variability. The seawater network, constructed from 9 seawater metagenomes, exhibited higher overall complexity, including more nodes (141 vs. 80), substantially more edges (1,530 vs. 340), higher edge density (0.155 vs. 0.108) and a higher average degree (21.70 vs. 8.50), indicating more densely connected associations in the water column. A small number of negative correlations (36 edges) were observed in the seawater network, whereas the sediment network consisted exclusively of positive associations.

**Fig. 5.**
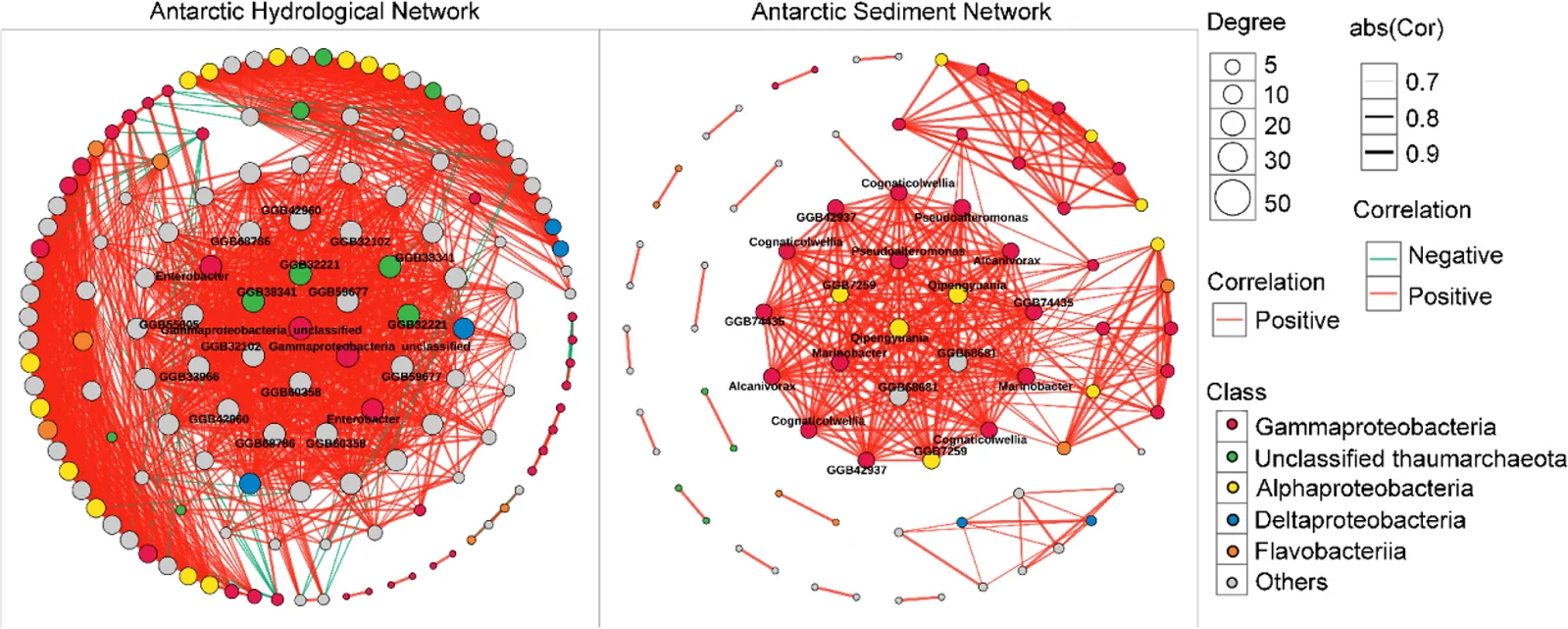
Co-occurrence networks of microbial ASVs in Antarctic seawater (left) and sediment (right), annotated at the genus and class levels. Networks were constructed at the ASV level using abundance profiles derived from the paired-end reads. Each node represents an ASV, labelled by its corresponding genus, with the top 20 genera (ranked by node degree) highlighted. Node size is proportional to degree (number of co-occurring connections). Node fill colour reflects the class-level taxonomic annotation, with colour assigned to the top abundant classes ranked by total relative abundance across all samples; remaining classes are grouped as others. Edge colour indicates the correlation type between ASVs: red for positive and green for negative associations. Edge transparency reflects the correlation strength (|*ρ*|).

Network topology metrics further highlighted contrasting organizational features. The seawater network displayed a longer average path length (3.46 vs. 1.30) and larger diameter (10.14 vs. 2.66), suggesting more extended interaction pathways and a potentially more hierarchical structure. In contrast, the sediment network, constructed from 13 publicly available Antarctic sediment metagenomes collected from comparable bathyal depths (~412–765 m), appeared more compact and modular, with a slightly higher clustering coefficient (0.868 vs. 0.846) and a greater number of modules (19 vs. 7), indicating stronger local connectivity and a more fragmented organization. Both networks exhibited zero edge connectivity, suggesting potential sensitivity to the loss of highly connected nodes.

Differences were also observed in the identity and topological roles of highly connected taxa. In seawater, nodes with high degree and betweenness centrality were predominantly unclassified lineages, including *Gammaproteobacteria*_unclassified and several CFGB-designated taxa such as GGB32102, GGB32221 and GGB60358. Some of these taxa exhibited high betweenness despite relatively low abundance, suggesting that low-abundance taxa may occupy structurally important positions within microbial networks, consistent with previous observations of rare taxa functioning as network hubs [63, 64]. In contrast, sediment networks were dominated by well-characterized genera such as *Qipengyuania*, *Marinobacter* and *Pseudoalteromonas*. These taxa exhibited relatively high degree but generally lower betweenness, indicating roles more confined to local modules rather than global connectivity.

Overall, these topological differences suggest distinct patterns of network organization between pelagic and benthic microbial communities. The higher connectivity and presence of negative correlations in seawater may reflect more complex or dynamic association patterns, potentially involving both positive and negative interactions as reported in other marine systems [65]. In contrast, the more modular structure and predominance of positive associations in sediment networks may be consistent with locally constrained and potentially cooperative interactions under broad spatial coverage across Antarctic sites, as suggested in previous studies of energy-limited environments [58, 66]. Nevertheless, these ecological interpretations should be considered with caution. The seawater and sediment datasets were not derived from strictly matched sampling locations, and detailed physicochemical metadata were not consistently available across all datasets. Given that microbial co-occurrence patterns are highly sensitive to environmental gradients such as depth, oxygen availability and organic matter content, the observed differences between networks may partly reflect underlying environmental heterogeneity rather than exclusively habitat-specific ecological strategies. Future studies incorporating matched environmental measurements across Antarctic habitats will be essential for disentangling habitat-specific ecological effects from environmental heterogeneity and for clarifying how local factors, including sea-ice dynamics, organic matter availability and water mass properties, shape microbial interaction networks. Therefore, the co-occurrence analysis presented here should be viewed as a descriptive and hypothesis-generating framework rather than definitive evidence of ecological interactions. Future studies incorporating matched sampling designs and environmental metadata will be necessary to further disentangle the relative contributions of habitat and environmental drivers to microbial network structure. Notably, taxa identified as highly connected in sediment networks, including *Qipengyuania*, *Marinobacter* and *Pseudoalteromonas*, have been previously associated with organic matter degradation and metabolic versatility in marine sediments [67–69], supporting their potential ecological relevance while acknowledging the limitations of inference from co-occurrence alone.

## Conclusions

By integrating metagenomic data of the Antarctic deep-sea microbiome, we expanded the reference microbial genomes from Antarctic deep-sea sediment and seawater and constructed an integrated microbial genome database. These data provided a foundation for further functional characterizations and taxonomic assignments of microbial diversity, metabolism and ecological interactions in polar oceans. The reconstructed MAGs from deep-sea environments revealed distinct habitat-specific assemblages and extensive functional capacities, including genes for biogeochemical cycling, secondary metabolism, virulence-associated defence and cold adaptation. Co-occurrence analysis further demonstrated divergent interaction structures between seawater and sediments. Collectively, these findings expanded current knowledge of Antarctic microbial ecology and highlight the ecological resilience and biotechnological potential of uncultured taxa as reservoirs of novel enzymes, metabolites and stress-resistance traits.

## Supplementary material

10.1099/mgen.0.001847 Supplementary Material 1.

10.1099/mgen.0.001847 Supplementary Material 2.

## Abbreviations

AMP: antimicrobial peptide
ASV: amplicon sequence variant
BGC: biosynthetic gene cluster
CAZyme: carbohydrate-active enzyme
dsMDAO: deep-sea microbial dataset of the Antarctic Ocean
GH: glycoside hydrolase
MAG: metagenome-assembled genomes
NRPS: nonribosomal peptide synthetase
PCoA: principal coordinate analysis
PKS: polyketide synthase
VFDB: Virulence Factor Database
