# Supplementary material for: Deep-sea Microbial Dataset of the Antarctic Ocean (dsMDAO): A high-resolution deep-sea microbial dataset of the Antarctic Ocean reveals potential for taxonomic and functional diversity
Source: Microb Genom. 2026 Oct 5;12(10):001847. doi: 10.1099/mgen.0.001847 (PMC13638183; doi:10.1099/mgen.0.001847)
Supplement: Supplementary file 1 [file mgen-12-01847-s001.pdf]

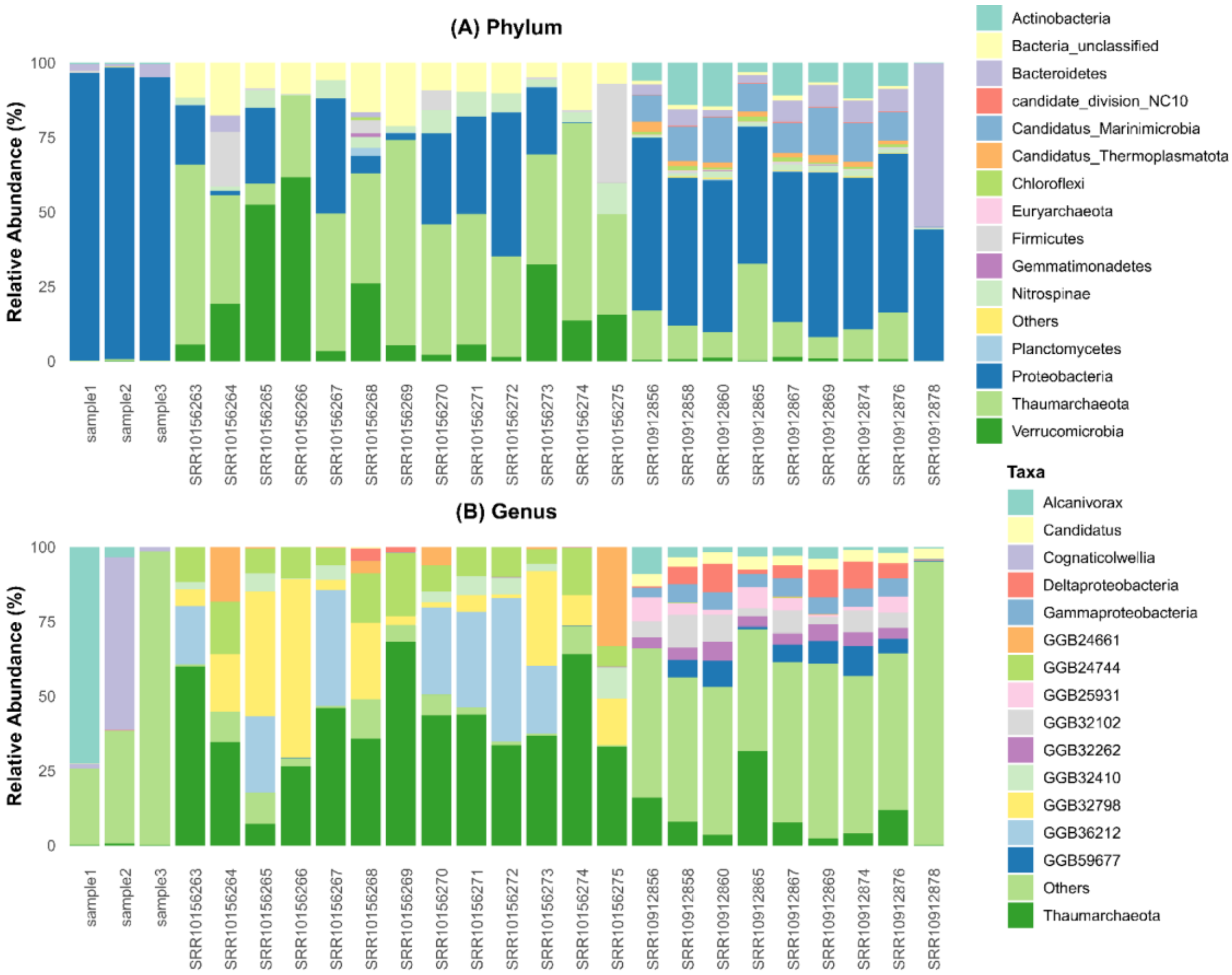

Figure S1. Taxonomic composition of microbial communities across the Antarctic deep-sea sediment (*n* = 16) and seawater samples (*n* = 9) at phylum and genus levels. The top panel (A) showed the relative abundance of the top15 microbial phyla across 25 samples, while the bottom panel (B) presented the top15 microbial genera. Taxonomic classification was performed using Kraken2, and abundance estimation was refined using Bracken. The sample set included 3 in situ sediment samples from Prydz Bay (sample 1-sample 3), 13 publicly available sediment metagenomes (SRR10156263-SRR10156275), and 9 publicly available seawater metagenomes (SRR10912856-SRR10912878).
